# Supplementary material for: A Bioluminescence-Based Ex Vivo Burn Wound Model for Real-Time Assessment of Novel Phage-Inspired Enzybiotics
Source: Pharmaceutics. 2022 Nov 22;14(12):2553. doi: 10.3390/pharmaceutics14122553 (PMC9781546; doi:10.3390/pharmaceutics14122553)
Supplement: Supplementary file 1 [file pharmaceutics-14-02553-s001.zip › pharmaceutics-2019780-supplementary.pdf]

Supplementary Figures

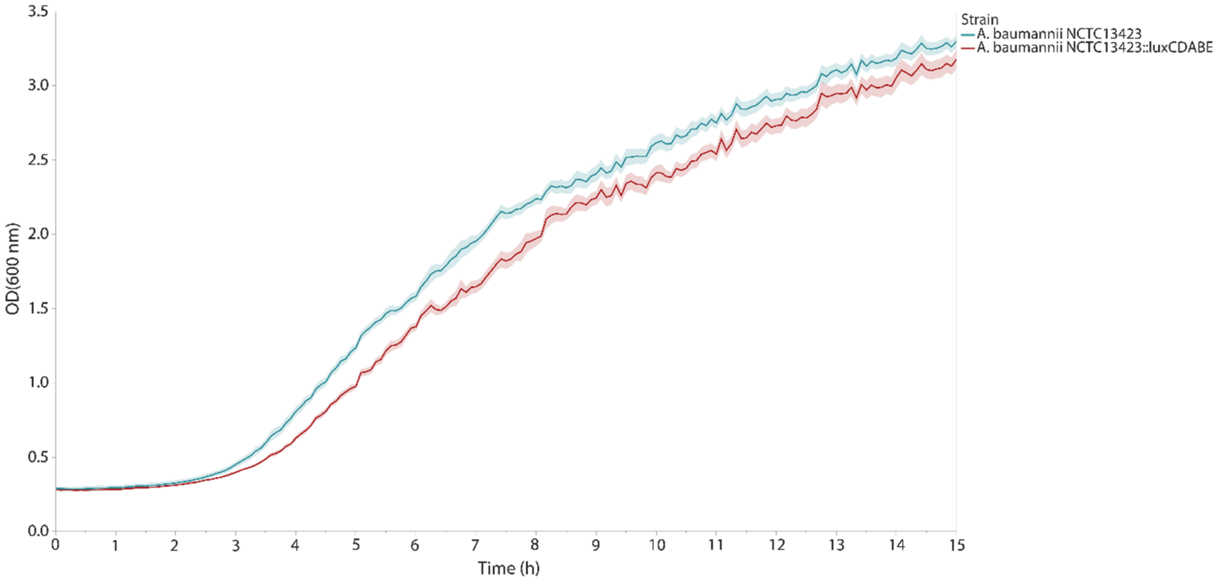

Figure S1: Growth curves of *A. baumannii* NCTC13423 and *A. baumannii* NCTC13423::*luxCDABE*. Overnight cultures of three biological replicates (n= 3) were diluted 100x and incubated at 30°C and shaking at 300 rpm). Every five minutes, optical density at 600 nm (OD_{600}) was measured on a Clariostar Plus (BMG Labtech, The Netherlands). Each biological replicate contained eight technical replicates. The error bands indicate one standard deviation from the mean of eight technical replicates of three biological replicates.

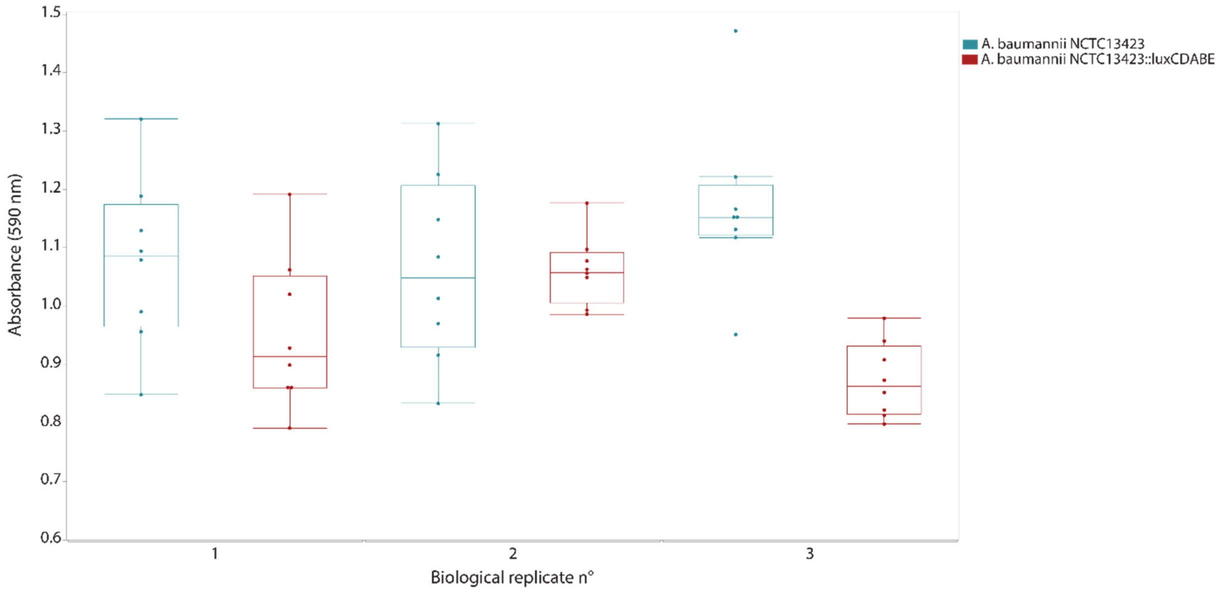

Figure S2: *In vitro* biofilm biomass of 24h old *A. baumannii* NCTC13423 biofilms. To verify whether *A. baumannii* NCTC13423 was still able to form biofilms upon *luxCDABE* insertion, an *in vitro* biofilm experiment was performed. Briefly, each overnight culture was diluted 100-fold in LB medium, after which 150 µL of this suspension was transferred to the wells of a Calgary Biofilm Device and incubated statically for 24h at 30°C (Ceri et al. 1999). Then, the peglids were washed with 175 µL sterile PBS to remove loose planktonic bacteria and stained with 200 µL of a 0.1 % (w/v) crystal violet solution (5 % (v/v) isopropanol (Sigma Aldrich), 5 % (v/v) methanol (Acros Organics, Belgium), 90 % (v/v) PBS) to quantify the biofilm biomass. Destaining was performed after washing three times in 200 µL PBS and a five-minute destaining step in 200 µL of a 30 % (v/v) acetic acid solution (Chem-Lab, Belgium). This graph displays three biological replicates with each eight technical replicates of a tagged and untagged strain. In each of the three replicates, the reporter strain was still able to establish a biofilm, albeit to a lesser extent as for example in the third replicate.

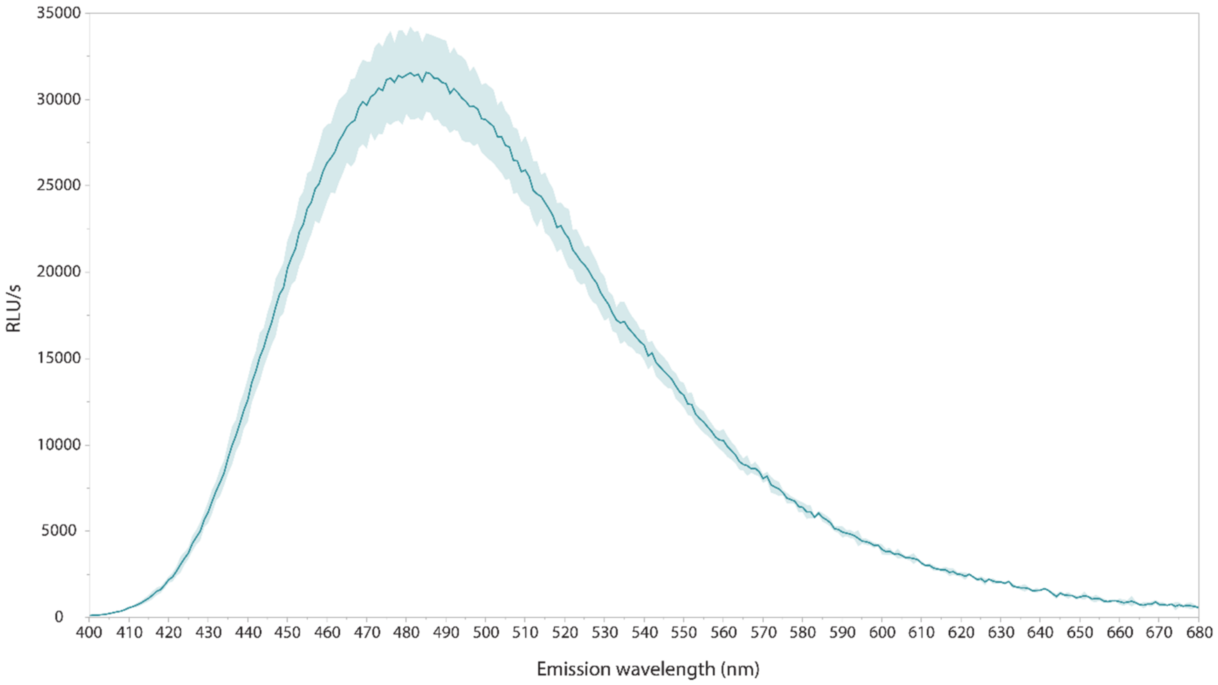

Figure S3: Emission spectrum of *A. baumannii* NCTC13423::*luxCDABE*. This graph describes the luminescence intensity (RLU/s) of a 1:100 diluted overnight culture for every wavelength ranging from 400 to 680 nm, corresponding to previously reported emission spectra of this reporter system. The error band was constructed using one standard deviation from the mean of three biological replicates (n = 3).

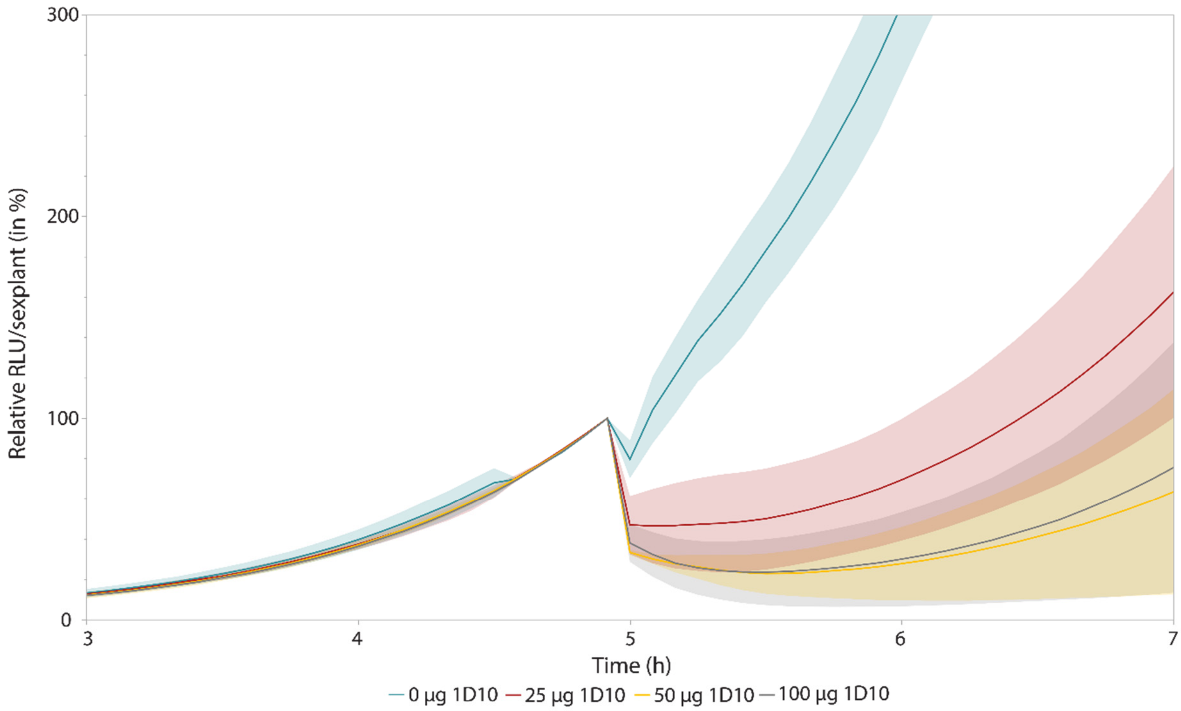

Figure S4: Relative bioluminescence intensity measurement to characterize the antibacterial effect of the engineered lysin 1D10. For every explant, the data was normalized for its value right before addition of the engineered lysin, so that this point corresponds to 100% growth. This then allows to quantify the average, relative drop in luminescence intensity indicating antibacterial performance. This is illustrated in Table 1. Every condition consists of three biological replicates (n = 3). The error bands indicate one standard deviation from the mean.

Supplementary Tables

Table S1: Comparison of logarithmic transformations of the RLU values measured from samples of uninfected explants (baseline) and infected explants for each time point. Each time point consists of three biological replicates (n = 3), and an asterisk indicates a significant difference for each measured time point between the signal and the baseline at significance level $\alpha = 0.05$ using a two sided Student's *t*-test.

Time point	Mean signal value (LOG10(RLU))	Mean baseline value (LOG10(RLU))	p-value
0 h	1.51 ± 0.0852	1.06 ± 0.0631	0.0039*
1 h	1.39 ± 0.0493	1.08 ± 0.0702	0.0008*
3 h	1.74 ± 0.0422	1.11 ± 0.0529	0.0001*
5 h	2.87 ± 0.0207	1.10 ± 0.0648	< 0.0001*
7 h	3.54 ± 0.137	1.17 ± 0.0482	0.0004*
9 h	4.14 ± 0.0458	1.12 ± 0.289	< 0.0001*
12 h	4.80 ± 0.110	1.20 ± 0.0918	< 0.0001*
24 h	4.83 ± 0.273	1.13 ± 0.0412	0.0008*

Table S2: Primers used in this work.

Name	Sequence (5'-3')	Used for
CmR_F	GCGGGTCTCGATGGAGAAAAAAATCACTGG	**Construction of pBGlux_CmR**: amplification of CmR resistance gene from pLemo
CmR_R	ATAGGTCTCATTACGCCCCGCCCTG	**Construction of pBGlux_CmR**: amplification of CmR resistance gene from pLemo
pBG13_Abmark_F	GCGGGTCTCAGTAACAATTCGTTCAAGCCG	**Construction of pBGlux_CmR**: inverse PCR allowing exchange of selectable marker using Type IIs-based cloning
pBG13_Abmark_R	GTCGGTCTCACCATCGTTGCTGCTCCATAAG	**Construction of pBGlux_CmR**: inverse PCR allowing exchange of selectable marker using Type IIs-based cloning
GA_luxCDABE_F	CATGCTAAGGAGGTTTTCTAATGACTAAAAA AATTTCATTCATTATTAACGGCCAGG	**Construction of pBGlux and pBGlux_CmR**: Tail-PCR including overlapping sequences used for Gibson assembly when isolating the *luxCDABE* operon from pSEVA426
GA_luxCDABE_R	GTACCGAGCTCGAATTCTCAACTATCAAACG CTTCGGTTAAGC	**Construction of pBGlux and pBGlux_CmR**: Tail-PCR including overlapping sequences used for Gibson assembly when isolating the *luxCDABE* operon from pSEVA426
GA_pBG_F	CCGAAGCGTTTGATAGTTGAGAATTCGAGCT CGGTACCCG	**Construction of pBGlux and pBGlux_CmR**: Inverse PCR including overlapping sequences used for Gibson assembly.
GA_pBG_R	GAATGAAATTTTTTTAGTCATTAGAAAACCTC CTTAGCATGATTAAGATGTTTC	**Construction of pBGlux and pBGlux_CmR**: Tail-PCR including overlapping sequences used for Gibson assembly.
ABglms_v2_F	TTTGCTGATGAAAATAGCGG	**Verification of insertion in *A. baumannii* NCTC13423**
Tn7_R	CACAGCATAACTGGACTGATTTC	**Verification of insertion in *A. baumannii* NCTC13423**

Table S3: Used DNA vectors and their selectable markers used in this work

Vector	Selectable marker
pBG13	Kanamycin[1] (50 µg/mL)
pBGlux	Kanamycin (50 µg/mL)
pBGlux_CmR	Chloramphenicol[2] (30 µg/mL or 90 µg/mL*)
pRK_2013	Kanamycin (25 µg/mL)

[1]Thermo Fischer Scientific [2]Sigma Aldrich, USA
*30 µg/mL was used in all *E. coli* strains; 90 µg/mL was used for selection in *A. baumannii* NCTC13423
